# Reply to Daly, J.J. Comment on “Chow, J.W.; Stokic, D.S. Longitudinal Changes in Temporospatial Gait Characteristics during the First Year Post-Stroke. *Brain Sci*. 2021, *11*, 1648”

**DOI:** 10.3390/brainsci12080997

**Published:** 2022-07-28

**Authors:** John W. Chow, Dobrivoje S. Stokic

**Affiliations:** Center for Neuroscience and Neurological Recovery, Methodist Rehabilitation Center, Jackson, MS 39216, USA; dstokic@mmrcrehab.org

This commentary [1] raises two points, the first of which is not quite relevant to our study [2]. We would like to emphasize that we made it clear up front that our study was descriptive (rather than mechanistic). Nowhere in the manuscript did we imply that the reported temporospatial gait parameters are “true mechanistic measures” and that we used them to “give insight into the true mechanism of change” or “understand the meaning underlying any gait speed changes”. Thus, we will put the first point to rest.

The second point relates to our secondary (exploratory) study objective. Whereas the commentary claims that we made a “false statement in the results”, this is far from the truth. All the reported results follow the procedures described in the Methods and are factually correct.

Regarding the possible violation of the t-test assumptions due to the dependency of observations (gait cycles), we recognize that the subsequent gait cycles within a walk trial can be considered dependent. However, this concern is lessened by the known stride-to-stride variability in gait parameters (including stride speed) in the stroke population (e.g., [3,4]). Moreover, subsequent walk trials are considered independent, leading to the recommendation that independent test procedures are appropriate for analyzing single-subject data [5]. Thus, although a valid concern, aggregating a variable number of gait cycles from multiple walk trials likely resulted in fewer dependent observations than initially suspected.

The bottom line is that the commentary insists on relying only on the minimal detectable change (MDC) approach for determining individual changes in gait speed between the 6- and 12-month evaluations, according to the published criteria for different speed categories [6]. Indeed, we did not object to including this approach in the latest revision nor did we express a preference against or in favor of it. It is puzzling, however, that the commentary did not provide any counterarguments to the limitations of the advocated MDC approach, as addressed in our Discussion, let alone several study limitations already pointed out by the authors themselves [6] (pp. 125–126).

In the interest of full transparency, it should be made known that the two points brought up correspond to those raised in the additional review of our manuscript that was communicated to us after the manuscript was officially accepted and published in Brain Sciences, a rather uncommon practice. This notwithstanding, we have been responsive to the relevant comments and again have revised our manuscript in a way that does not substantially alter the content of the already published article, which has been accessed over 550 times at the time of this writing, hence the content additions rather than the deletions [2]. We believe that our response to the additional review was the most prudent approach given the circumstances and was also in the best interest of the journal readership.

In closing, nothing is misleading in our article. Everything is presented transparently, and the readers are provided will all the necessary information to interpret the findings for themselves. Most of all, we are confident that the readers will not miss the forest for the trees.
